# Effects of BMP-2 Delivery in Calcium Phosphate Bone Graft Materials with Different Compositions on Bone Regeneration

**DOI:** 10.3390/ma9110954

**Published:** 2016-11-23

**Authors:** Jin-Chul Park, Eun-Bin Bae, Se-Eun Kim, So-Yun Kim, Kyung-Hee Choi, Jae-Won Choi, Ji-Hyeon Bae, Jae-Jun Ryu, Jung-Bo Huh

**Affiliations:** 1 Department of Dentistry, School of Medicine, Korea University, Seoul 02841, Korea; jinchol09@hanmail.net; 2 Department of Prosthodontics, BK21 PLUS Project, School of Dentistry, Pusan National University, Yangsan 50612, Korea; 0228dmqls@hanmail.net (E.-B.B.); won9180@hanmail.net (J.-W.C.); say0739@daum.net (J.-H.B.); 3 Department of Veterinary Surgery, College of Veterinary Medicine, Chonnam National University, Gwangju 61186, Korea; sen0223@gmail.com; 4 School of Dentistry, Pusan National University, Yangsan 50612, Korea; annasoyunkim@gmail.com; 5 Tissue Biotech Institute, Cowellmedi Co., Ltd., Busan 46986, Korea; ckh@cowellmedi.com

**Keywords:** alloplast, bone substitutes, biphasic calcium phosphate, calcium pyrophosphate, rhBMP-2

## Abstract

This study was undertaken to investigate the effect of loading rhBMP-2 onto biphasic calcium phosphate (BCP) and calcium pyrophosphate (CPP) on bone regeneration, and to examine the efficacies of BCP and CPP as rhBMP-2 carriers. Specimens were divided into the BCP, CPP, BCP/BMP, and CPP/BMP groups; BCP and CPP were in granules and not coated with rhBMP-2. BCP/BMP and CPP/BMP were prepared as discs, which were treated with rhBMP-2 and collagen. Physical and biological features were investigated using in-vitro and in-vivo tests. New bone area percentages (%) in the BCP/BMP and CPP/BMP groups were significantly greater than in the BCP and CPP groups. At weeks 4 and 8 post-implantation, CPP/BMP showed the most new bone growth. Within the limitations of this study, treatment of BCP and CPP with rhBMP-2 significantly enhanced bone regeneration. CPP was found to be a suitable carrier for rhBMP-2.

## 1. Introduction

The use of biomaterials for bone regeneration has been widely utilized by dental bone augmentation procedures such as extraction socket defect grafting, sinus augmentation, and ridge augmentation [1]. Bone graft biomaterials should possess the properties of osteoconduction and osteoinduction to promote the formation of new bone [2]. Autogenic bone grafting has been traditionally used for defect reconstruction, but more recently a variety of graft materials, such as allogenic, xenogenic, and synthetic bones, have been introduced [3,4]. Autogenic bone is the most ideal graft material due to its outstanding biocompatibility, bone formation ability, osteoinductivity, and osteoconductivity. However, available amounts are limited, its resorption pattern is difficult to predict, and the additional surgery for harvesting causes patient discomfort and introduces risks of possible complications [3,4,5]. Allogenic and xenogenic bones are obtained from corpses and animals, respectively, and thus, no harvesting is required and availability is not an issue, but immune rejection and cross infection are possible [6]. Accordingly, alloplast grafting is becoming increasingly popular, and calcium phosphates are commonly used because their chemical compositions are similar to that of natural bone [7,8,9,10,11].

Hydroxyapatite (Ca_5_(PO_4_)_3_(OH) or HA), *β*-tricalcium phosphate (Ca_3_(PO_4_)_2_ or *β*-TCP), and calcium pyrophosphate (Ca_2_P_2_O_7_ or CPP) are representative of calcium phosphates used as biological graft materials [12]. HA is widely used due to its excellent biocompatibility and osteoconductivity in dentistry [12,13], but its biodegradability is poor, as such, the graft material remains in defects for a long period of time and ultimately blocks new bone formation [14,15]. The effort to improve the degradation rate of HA through granting porosity faces a limitation since it results in weaker compressive strength unsuitable for load-bearing application [16]. The *β*-TCP has a faster in vivo degradation rate than HA but its mechanical properties are inferior, and thus, biphasic calcium phosphate (BCP; a mixture of HA and *β*-TCP) is used [3,17]. On the other hand, the degradation rate of calcium pyrophosphate (CPP) is faster than those of *β*-TCP and HA, and positive results have been reported in terms of space maintenance when CPP was used alone [18,19]. Consequently, CPP is considered a near ideal alloplast. The outstanding biocompatibility of CPP with bone tissue has been verified in-vitro and in-vivo [10,20]. In one study, in which CPP was applied to tibial metaphyses of rats and rabbits, the biodegradability and new bone formation ability of CPP were found to be comparable to those of HA [8,9,10]. An ideal bone substitute plays a role as a focus for new bone formation during resorption [21]; when CPP is grafted into a bone defect, new bone formation occurs underneath the graft [8,10,11]. Kitsug et al. [9] observed direct contact between CPP and bone using transmission electron microscopy (TEM), and no interposition of soft tissue. Despite of the reported advantages of CPP, it has not been widely used as a bone substitute because when CPP degrades it releases pyrophosphate (P_2_O_7_^4−^) which inhibits HA formation [22]. However, recent studies have shown alkaline phosphatase (ALP), which is secreted by osteoblasts, hydrolyzes pyrophosphate and prevents it inhibiting HA formation, and that the phosphate (PO_4_^−^) produced fosters mineralization. Thus, it appears CPP can be utilized as a bone substitute [23].

The use of synthetic bone in clinical practice is limited by lack of osteoinductivity [24]. Osteoinductive proteins, such as recombinant human bone morphogenetic protein-2 (rhBMP-2), promote the differentiation of mesenchymal stem cells (MSCs) and pre-osteoblast into osteoblasts and trigger the migration of osteoblasts [25,26]. Recent clinical and histological studies have demonstrated the addition of rhBMP-2 during bone grafting improves results [27,28], and another study reported that injection of rhBMP-2 induced orthotopic and ectopic bone formation [29]. Furthermore, it has been shown rhBMP-2 presents a low risk of adverse immune system reactions [30].

Some studies have confirmed rhBMP-2 has a favorable effect on new bone formation when used in conjunction with BCP or HA for the rehabilitation of alveolar defects [24,31]. However, no study has yet investigated the rhBMP-2/CPP combination even though CPP degrades faster than *β*-TCP or HA and has a porous architecture that could be used to deliver rhBMP-2 [18]. In the present study, we investigated the effect of loading rhBMP-2 into calcium phosphate bone graft materials with different compositions (BCP and CPP) on bone regeneration and evaluated the feasibility of using BCP or CPP as rhBMP-2 carriers.

## 2. Results

### 2.1. Observations of Surface Morphology

**Figure 1:**
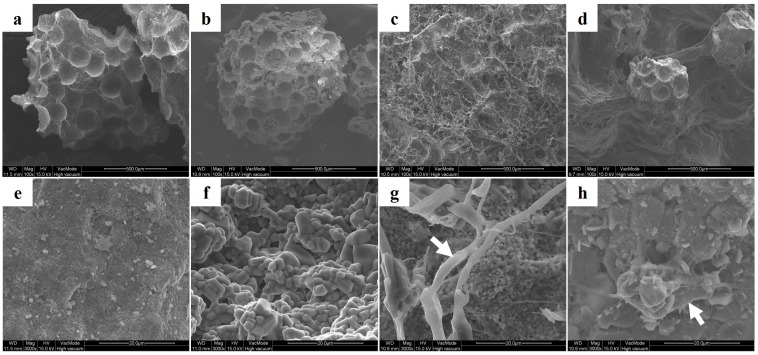
Scanning electron microscopic photographs of (**a**,**e**) biphasic calcium phosphate (BCP) granules; (**b**,**f**) calcium pyrophosphate (CPP) granules; (**c**,**g**) a BCP/BMP disc; (**d**,**h**) a CPP/BMP disc. White arrow: rhBMP-2 coated collagen (Original magnification ×100 for **a** to **d** and ×3000 for **e** to **h**).

SEM analysis was used to determine pore sizes and examine surface morphologies (Figure 1). BCP had a pore size of 0.1 µm–1 µm (Figure 1e), whereas CPP had a pore size of 10 µm–100 µm (Figure 1f). rhBMP-2 with collagen covered the surfaces of BCP/BMP (Figure 1g) and CPP/BMP (Figure 1h).

### 2.2. Release Kinetics of RhBMP-2

**Figure 2:**
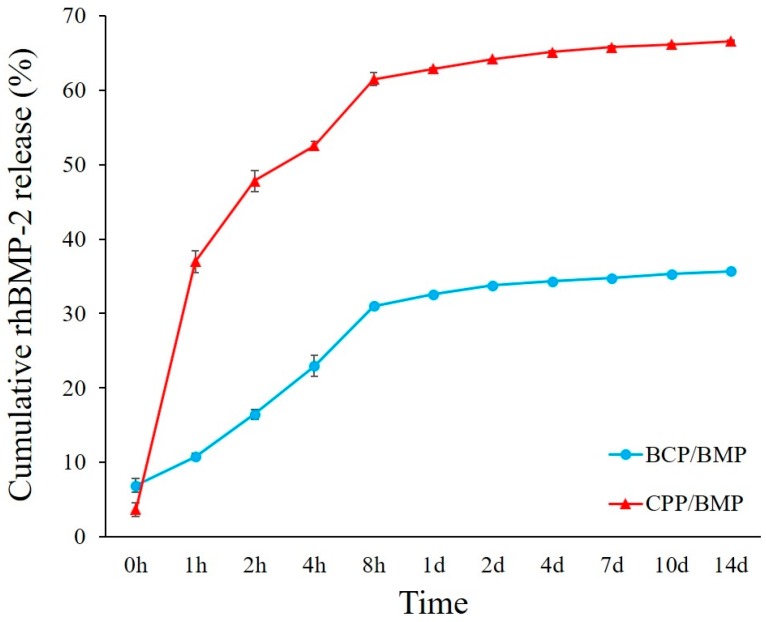
Release kinetics of rhBMP-2. CPP/BMP released double the amount of rhBMP-2 than BCP/BMP.

In terms of the accumulated amounts of rhBMP-2 released, the release kinetics of rhBMP-2 from BCP/BMP and CPP/BMP are shown in Figure 2. BCP/BMP, which contained 0.4 µg of rhBMP-2, released 142.68 ng of rhBMP-2, and CPP/BMP released 299.57 ng in 14 days. On the first day, 90% of the total amount of rhBMP-2 released in 2 weeks was rapidly discharged from both BCP/BMP and CPP/BMP, with values of 130.28 ng and 282.85 ng, respectively.

### 2.3. Observation of Cell Attachment

**Figure 3:**
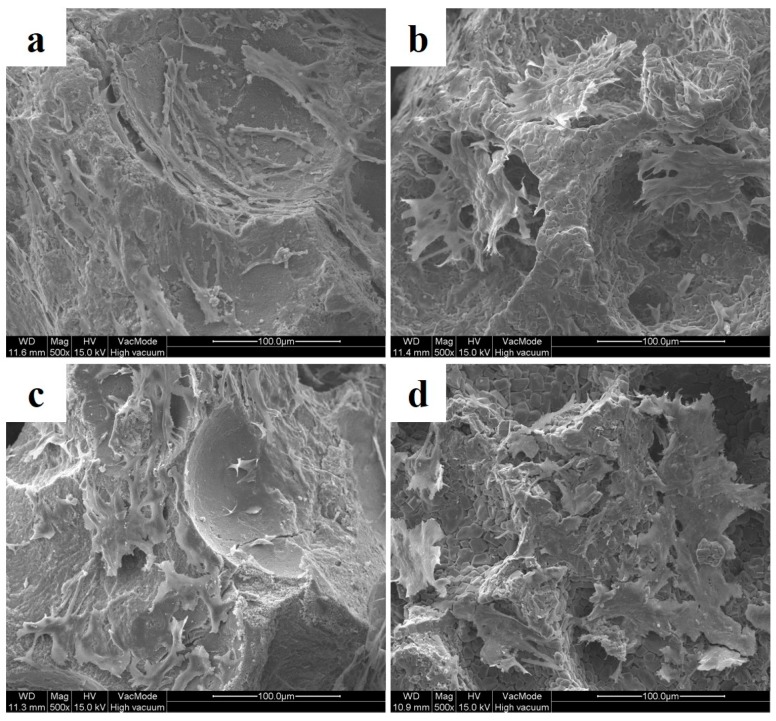
SEM photographs of graft material surfaces after culture with C2C12 for 14 days. (**a**) BCP granule; (**b**) CPP granule; (**c**) BCP/BMP granule; and (**d**) CPP/BMP granule (original magnification ×500).

In order to observe cell attachment profiles on the different graft materials, C2C12 cells were introduced to each experimental group and cultured for 14 days. In BCP and BCP/BMP, attached cells were spread over surfaces (Figure 3a,c), whereas in CPP and CPP/BMP, cells formed colonies (Figure 3b,d).

### 2.4. Measurement of Cell Proliferation

**Figure 4:**
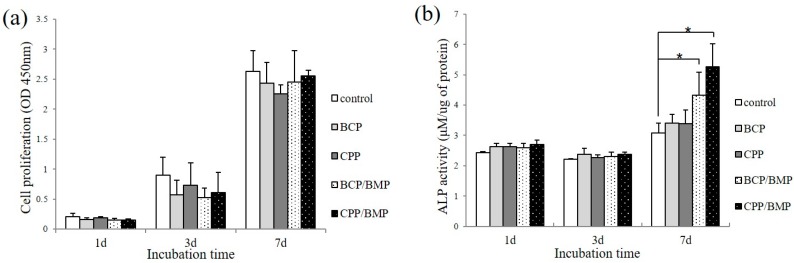
(**a**) Proliferation and (**b**) Alkaline phosphatase (ALP) activity of myoblast cells (C2C12 cells) grown on BCP, CPP, BCP/BMP or CPP/BMP after 1, 3, or 7 days of incubation. The symbol ‘*’ indicates significantly different versus the control (*p* < 0.05).

Cell proliferations in the control and experimental cultures are shown in Figure 4a. At day 1, 3, and 7, no difference was observed between any of the groups (Figure 4a), indicating none of the materials were cytotoxic.

### 2.5. Measurement of Alkaline Phosphatase (ALP) Activity

The levels of ALP activity in control and experimental cultures are shown in Figure 4b. On days 1 and 3, ALP activities were similar in the experimental and control groups. However, on day 7, BCP/BMP showed significantly higher ALP activity than the control (*p* < 0.05), and CPP/BMP yielded significantly higher activity than the control, BCP, and CPP groups (*p* < 0.05).

### 2.6. Histologic Findings in Animal Study

**Figure 5:**
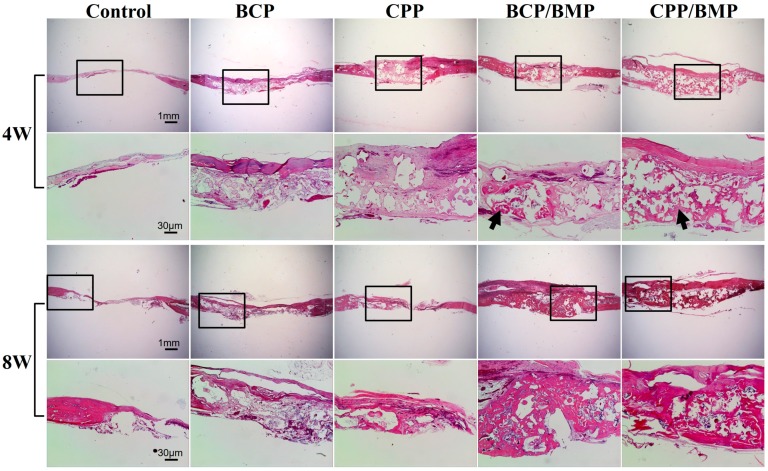
Hematoxylin and eosin staining of histological sections of defect sites at 4 and 8 weeks’ post-implantation. Black arrow; newly formed bone (original magnification: ×12.5 for rows 1 and 3, and ×40 for rows 2 and 4).

Images of histological sections of the control and experimental groups were prepared at week 4 and week 8 post-implantation (Figure 5). The control showed minor new bone formation at both times and a small amount of fibrous tissue. Both BCP and CPP exhibited fibrous tissue around graft materials on week 4, and small amounts of new bone formation on week 8. BCP/BMP and CPP/BMP displayed a small amount of new bone formation around the graft materials on week 4 and more new bone formation on week 8.

### 2.7. Histometric Findings in Animal Study

**Figure 6:**
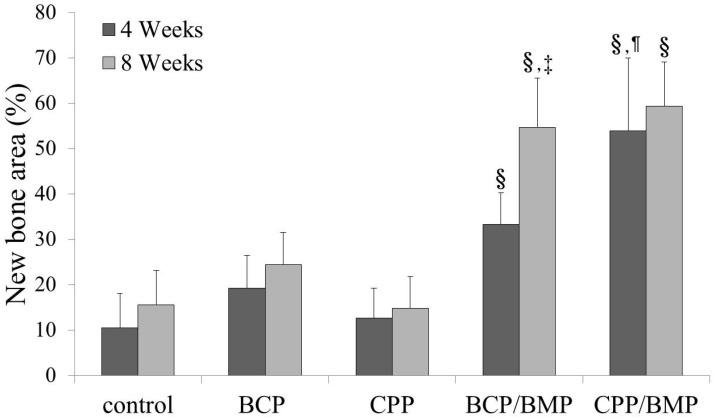
New bone area percentages at 4 and 8 weeks’ post-implantation. No significant differences were found between the control, BCP, and CPP groups at 4 or 8 weeks. The symbol ‘§’ indicates significantly higher percentage versus the control, BCP, and CPP groups at the indicated time (*p* < 0.05). The symbol ‘¶’ indicates significantly higher percentage versus BCP/BMP group at the indicated time (*p* < 0.05). ‘‡’ indicates significantly higher percentage versus the same group at 4 weeks (*p* < 0.05).

New bone densities (%) are shown in Figure 6. On weeks 4 and 8, the BCP and CPP groups were not significantly different than the control group (*p* > 0.05), but the experimental groups showed significantly greater new bone densities than the control, BCP, and CPP groups (*p* < 0.05). As compared to new bone densities measured at week 4, the control, BCP, CPP, and CPP/BMP groups did not exhibit a significant increase at week 8 (*p* > 0.05). However, the BCP/BMP group demonstrated a significant increase in new bone density at week 8 (*p* < 0.05). New bone formation was significantly greater in the CPP/BMP group than in the BCP/BMP group at 4 weeks (*p* < 0.05) but not at 8 weeks (*p* > 0.05).

## 3. Discussion

An ideal bone graft material should have sufficient strength and stability as a scaffold to promote new bone, function as a channel for osteoinductive materials, degrade optimally, and be replaced by new bone [32,33]. As reported previously, CPP induces a biological response similar to those of HA and has excellent biodegradability. Sun et al. [20] verified an outstanding bioactivity of CPP by demonstrating the oral administration of CPP to ovariectomized rats restricted an increase in bony trabecular porosity and promoted bone mineralization in long bones. Moreover, physical properties including particle size, crystallinity, porosity, and surface roughness, and chemical properties including Ca/P ratio and pH affect the bioactivities and biodegradabilities of bone graft materials. Furthermore, several studies have investigated the effect of different Ca/P ratios on bone regeneration [34,35]. In the present study, CPP with Ca/P ratio of 1 was compared with BCP with Ca/P ratio of 1.55, the most common graft material in clinical practice. In recent years, numerous studies have incorporated growth factors, such as, rhBMP-2, with various bone graft biomaterials to enhance bone regeneration [24]. The bone regeneration capacity of rhBMP-2 is dependent on carrier type [36,37], and a good carrier should load protein easily, secure a space for regeneration, and exhibit bioabsorbable and bioactive degradation. In the present study, two alloplasts, BCP and CPP, were loaded with rhBMP-2 to examine their feasibilities as compatible rhBMP-2 carriers. 

The two bone graft materials examined in the present study had rhBMP-2 release behaviors similar to those reported by Boyne et al [38], that is, BCP/BMP and CPP/BMP released more than 90% of their total release amounts within one day, and these rapid releases seemed to be responsible for the ineffectiveness of rhBMP-2 to induce new bone formation in clinical applications. To facilitate the sustained release of rhBMP-2, Huh et al. [39] attempted to chemically immobilize rhBMP-2 to DOPA-heparin on xenogenous bone. However, the chemical approach is challenging clinically and potential chemical toxicity restricts its clinical application. Accordingly, in the present study, we used a physical coating technique and avoided the use of any chemical functional group. Even though bone graft materials were coated physically, the different graft materials exhibited different release rate patterns, that is, CPP/BMP showed a higher accumulation rate than BCP/BMP, which was probably due to enhanced rhBMP-2 release caused by the higher degradation rate of CPP.

Numerous studies reported that bone substitute surface roughness affects cell adhesion and morphology [40,41]. Using SEM images of C2C12 cells, we confirmed that cell morphologies depended on bone substitute surface roughness rather than on the presence of growth factor in the BCP and BCP/BMP groups and in the CPP and CPP/BMP groups, but BCP and CPP containing groups differed. Furthermore, C2C12 cells in CPP proliferated more than in BCP, but the difference was not significant. Such difference in proliferation are due to the preference of cells for a rough surface, which is consistent with a report issued by Deligianni et al. [40].

As observed through histometric analysis, CPP/BMP exhibited significantly greater new bone formation than BCP/BMP at 4 weeks’ post-implantation, and this fast degradation rate of CPP increased the rate of rhBMP-2 release, and better promoted an early stage of new bone formation. Alam et al. [34] demonstrated that rapidly degraded bone graft materials create microenvironments that favor new bone formation. However, at 8 weeks, CPP/BMP and BCP/BMP did not show significant difference. This outcome can be explained by the gradual progress in new bone formation of BCP/BMP which initially released less rhBMP-2. On the other hand, new bone formation was not promoted in the BCP and CPP groups.

In the present study, BCP/BMP and CPP/BMP induced new bone formation and did not induce inflammatory responses, which is indicative of their biocompatibilities and potential as suitable rhBMP-2 carriers. However, it should be borne in mind that this in-vivo study was conducted using a small number of samples and observations at 4 and 8 weeks’ post-implantation, and therefore, we suggest further larger-scale, more comprehensive longitudinal studies be conducted. Furthermore, due to its hydrophilicity, rhBMP-2 should be delivered to targeted sites, and thus, studies are also required to optimize the method of rhBMP-2 loading and to determine its optimal dose and concentration.

## 4. Materials and Methods

### 4.1. Preparation of Porous Calcium Phosphate Coated with RhBMP-2

**Figure 7:**
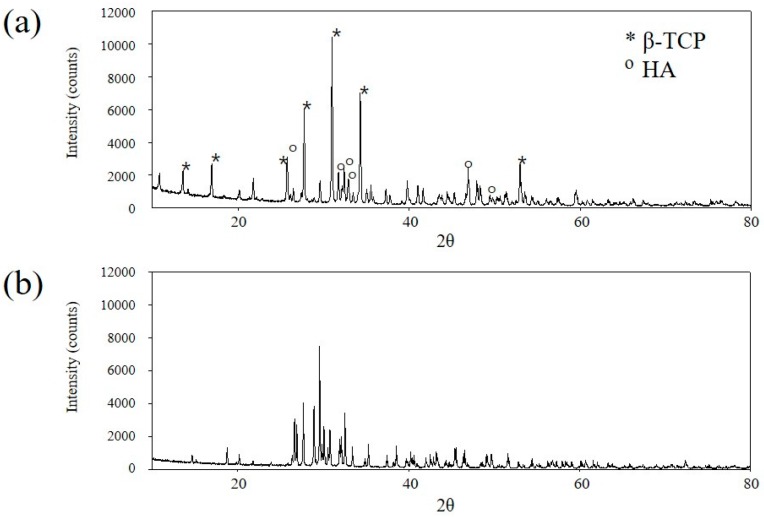
X-ray diffraction analysis (XRD) patterns of bone graft materials. (**a**) BCP containing HA(30): *β*-TCP(70); (**b**) CPP.

BCP granules (particle size 0.4 mm–1.0 mm, Cowellmedi, Busan, Korea) were prepared by sintering biphasic calcium phosphate powder (HA/*β*-TCP: 3/7; Figure 7a). CPP (particle size 0.4 mm–1.0 mm; Cowellmedi, Busan, Korea) was prepared by sintering dicalcium phosphate powder (Figure 7b). To prepare BCP/BMP and CPP/BMP, 0.3 mL of collagen (Cowellmedi, Busan, Korea) was added per 0.1 g of BCP or CPP granules. The mixture so obtained was freeze-dried to produce 1 mm thick discs of diameter 8 mm. Each disc was treated with 100 µL of rhBMP-2 solution, which was equivalent to a loading of 5 µg of rhBMP-2/ disc, and then freeze-dried.

### 4.2. Release Kinetics of rhBMP-2

BCP/BMP and CPP/BMP were placed to 1 mL of phosphate-buffered saline (pH = 7.4) containing 0.02% sodium azide and agitated in a shaking incubator at 37 °C and 200 rpm. Supernatants were collected, and a fresh 1 mL of PBS was added up to 14 days. Concentrations of rhBMP-2 in supernatants were determined using a human BMP-2 ELISA kit (Pepro Tech, Rocky Hill, NJ, USA) and absorbances were measured using a microplate reader at 495 nm.

### 4.3. Observations of Cell Attachment

In order to investigate cell attachments to different bone graft materials, a 48-well culture plate was prepared as mentioned in Section 2.3 using the same medium used for cell proliferation (medium was replaced every 3 days) and cultured under 5% CO_2_ at 37 °C for 14 days.

### 4.4. Measurement of Cell Proliferation

C2C12 myoblasts (1.5 × 10^4^ cells/well) were loaded into a 48-well culture plate, and then 0.01 g of BCP, CPP, BCP/BMP, or CPP/BMP was added. The plate was cultured under 5% CO_2_ at 37 °C for 1, 3, or 7 days. Dulbecco’s Modified Eagle’s Medium (DMEM) containing 10% FBS, 100 U/mL Penicillin, and 100 µg/mL Streptomycin was used as the culture medium. The control was prepared in an identical manner but without bone graft material. After culture, cells were counted using the Cell Counting Kit-8 (Dojindo, Tokyo, Japan).

### 4.5. Measurement of Alkaline Phosphatase Activity (ALP)

A 48-well culture plate containing C2C12 myoblasts was prepared and cultured as described above. For ALP activity measurements, medium was removed, and cells were separated with trypsin-EDTA and harvested by centrifugation. Cells were lysed using Lysis buffer (0.1% Triton X-100, 150 mM NaCl, 50 mM Tris, pH = 8.0) with sonication, and supernatants were collected by centrifugation. Protein concentrations were measured using Braford protein assay reagent at 595 nm, including that of the standard, BSA solution. *p*-Nitrophenyl phosphate (*p*-NPP) was used as the substrate for alkaline phosphatase. The mixture was allowed to react for 20 min, 100 µL of NaOH (1M, Daejungchem, Seoul, Korea) was added to stop the reaction, and then ALP activity was measured at 405 nm. ALP activity was recorded in µM/µg of protein.

### 4.6. In-Vivo Animal Study

Fifty Sprague-Dawley rats (male, weight 250 g–300 g) were used in the study. Animals were housed individually under standard laboratory conditions in plastic cages and had ad libitum access to water and standard laboratory pellets. Animal selection, management, the surgical protocol, and preparation were approved beforehand by the Ethics Committee on Animal Experimentation at the Korea Atomic Energy Institute (KAERI-IACUC-004).

**Figure 8:**
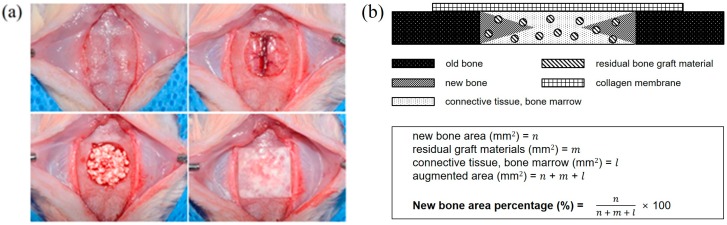
(**a**) Surgical procedures; (**b**) a schematic of the histometric analysis.

Surgical procedures were performed under general anesthesia induced by intramuscular injection. The surgical site was incised and a full-thickness flap was elevated. A standard, circular, transosseous defect of 8 mm in diameter was formed in the middle of calvarias using a trephine bur (3i Implant Innovations Inc., Palm Beach Garden, FL, USA). Treatments were performed after removing the trephined bony disk. Ten rats were assigned to each of the five study groups. Animals in the control group did not receive any treatment while animals in the four experiment groups (BCP, CPP, BCP/BMP, and CPP/BMP groups) received one specimen covered with a collagen membrane (GENOSS, Suwon, Korea) (Figure 8a). Five rats per group were sacrificed at 4 weeks’ post-implantation and the other five were sacrificed at 8 weeks.

After decalcifying calvarias with 14% EDTA, they were further decalcified using Rapid acid decal (Calci-clear rapid, National diagnostics, Atlanta, GA, USA). The middle of each paraffin-embedded calvarial defect was sectioned at 5 µm, and two of the most central sections from each block were stained with hematoxylin and eosin. To determine areas of new bone and of residual biomaterials, we used an image analysis program (Image-Pro Plus, Media Cybernetic, Silver Spring, MD, USA). New bone areas were calculated as percentages (refer to Figure 8b).

## 5. Conclusions

Bone morphogenetic protein-2 treated BCP or CPP significantly enhanced bone regeneration, and CPP was found to be a suitable carrier of rhBMP-2.

## Abbreviations

HA	Hydroxyapatite
*β*-TCP	*β*-tricalcium phosphate
BCP	Biphasic calcium phosphate
CPP	Calcium pyrophosphate
TEM	Transmission electron microscope
ALP	Alkaline phosphatase
RhBMP-2	Recombinant human bone morphogenetic protein-2
SEM	Scanning electron microscope
